# Psychological aspects in asthma: do psychological factors affect asthma management?

**DOI:** 10.1186/s40733-015-0007-1

**Published:** 2015-08-05

**Authors:** Ilaria Baiardini, Francesca Sicuro, Francesco Balbi, Giorgio Walter Canonica, Fulvio Braido

**Affiliations:** Allergy & Respiratory Diseases Department, Azienda Ospedaliera Universitaria IRCCS San Martino, Genoa, Italy

## Abstract

Despite the regular treatment with inhaled corticosteroids (ICS) or ICS plus long-acting beta2-agonists, permits to control de majority of asthmatics, a significant proportion of patients does not respond to this treatment.

This review was aimed to explore the role of psychological factors associated to the unsuccessful fulfilment of optimal levels of asthma control, especially in patients suffering from severe asthma. The results of a Medline search were 5510 articles addressed to different psychological key concepts, constructs and variables. This review will highlight how some selected psychological factors may have a burden on asthma management. Evidences are now available about the link between asthma (in terms of severity and control), some psychological aspects (subjective perception, alexithymia, coping style) and mental health (anxiety, depression). Taking into account this most probably bidirectional influence, a screening of mental symptoms and psychological aspects related to asthma, could lead to plan appropriate interventions to better control asthma and to improve the patient’s well-being.

## Introduction

It is now well recognized that the regular treatment with inhaled corticosteroids (ICS) or ICS plus long-acting beta2-agonists (LABA), permits to control the majority of asthmatics. However, a significant proportion of patients does not respond to this treatment [1, 2]. It has been estimated that severe asthma accounts for approximately 5–10 % of the total asthmatic population [3, 4]: this range most likely depends on changing definitions and criteria adopted in the past decades. Recently, a Task Force, supported by the European Respiratory Society (ERS) and American Thoracic Society (ATS) [5], proposed a revised definition of severe asthma in children ≥6 years and adults.

Severe asthma has been defined (5) as asthma which requires treatment with guidelines suggested medications for GINA steps 4–5 asthma (high dose ICS and LABA or leukotriene modifier/theophylline) for the previous year or systemic CS for ≥50 % of the previous year to prevent it from becoming “uncontrolled” or which remains “uncontrolled” despite this therapy (as described in Table 1). The definition of high dose inhaled corticosteroids (ICS) is age-specific.

**Table 1:** The definition of uncontrolled asthma [5]

Uncontrolled asthma defined as at least one of the following:
1) Poor symptom control: ACQ consistently >1.5, ACT <20 (or “not well controlled” according NAEPP/GINA guidelines);
2) Frequent severe exacerbations: two or more bursts of systemic CS (>3 days each) in the previous year;
3) Serious exacerbations: at least one hospitalisation, ICU stay or mechanical ventilation in the previous year;
4) Airflow limitation: after appropriate bronchodilator withhold FEV1 < 80 % predicted (in the face of reduced FEV1/FVC defined as less than the lower limit of normal);
Controlled asthma that worsens on tapering of these high doses of ICS or systemic CS (or additional biologics)
*

*GINA* Global initiative for asthma, *ICS* Inhaled corticosteroids, *LABA*, Long-acting beta2-agonists, *CS* Corticosteroids, *ACQ* Asthma control questionnaire, *ACT* Asthma control test, *NAEPP* National asthma education and prevention program, *ICU* Intensive care unit, *FEV1* Forced expiratory volume in the first second, *FVC* Forced vital capacity

The aim of asthma therapy is to reach and maintain the disease control, minimizing symptoms, daily activity limitation, and the risks for life-threatening exacerbations and long-term morbidity [6]. As available population-based studies clearly illustrate, in a large portion of asthma patients a satisfactory level of disease control is not achieved. In the Asthma Insights and Reality in Europe (AIRE) study, involving more than 2,800 subjects living in different European countries, more than 50 % of the interviewees exhibited daytime symptoms and one out of three respondents experienced asthma-related sleep problems [7]. In the International Asthma Patient Insight Research (INSPIRE) study 3,415 treated asthma adults were interviewed by telephone [8], nearly 74 % used a short-acting bronchodilator every day, and 51 % had at least one exacerbation that required medical treatment in the past year.

An incomplete success of asthma treatment may depend on the presence and interaction of different causes related to the disease itself (i.e. triggers, comorbidities, asthma phenotype), the treatment (i.e. inadequate treatment, inadequate inhaler device), the patient (i.e. socio-demographic factors, adherence, knowledge), the physician (i.e. lack of consciousness and familiarity about guidelines) [9]. Nevertheless, different psychological factors play a primary role in daily asthma management. The ideas that the patient has regarding asthma, the impact of the disease on daily life, the subjective interpretation of symptoms, and therapeutic adherence can be significantly influenced by psychological aspects. Moreover, the presence of the disease itself may have an impact on patients’ affective sphere, representing an obstacle to an optimal disease management. It is now recognized that asthma can influence mental health and vice versa [10, 11], and that a link exist between psychological functioning and level of asthma control [12].

The purpose of this review is to explore how different psychological aspects may be associated to the unsuccessful fulfilment of optimal levels of asthma control, especially in patients suffering from severe asthma.

To address this issue, Medline searches were performed by pairing the word ‘asthma’ with the following keywords: psychological factors, psychological disorders, and psychological characteristics. The results of our search were 5510 articles addressed to different psychological key concepts, constructs and variables. This review will highlight how some selected psychological factors may have a burden on asthma management.

## Review

### Specific psychological factors that likely affect asthma experience and management

Symptoms’ subjective perception, alexithymia, coping strategies, depression and anxiety are the psychological factors that, in cognitive or in emotional dimensions, are most involved in Asthma experience and management.

The personal report of disease, the difficulty in recognizing feelings and sensations, the ability to solve problems and tolerate stress, the presence of emotional and mental health disorders, can influence the success of the treatment and the prognosis of the disease.

The relationship between each dimension and the experience of Asthma will be analyzed in detail.

### Symptoms’ subjective perception

Patients’ report of symptoms constitutes an important factor in determining the diagnosis of asthma and in influencing the treatment plan. However, self-reported symptoms poorly correlate with pulmonary function measures and the perception is not necessarily linearly related to the sensory input: patients may exhibit limited disease severity, but describe high levels of dyspnea, or vice versa. From 15 to 60 % of patients [13], depending on the measurements used, report difficulty in recognizing their own degree of airway obstruction. Poor symptom perception may result in a significant overuse of reliever medication, irrespective of lung function, whereas under-perception presents the potential risk of delayed treatment. In a 2-year follow-up, patients with both increased and decreased symptom perception exhibited a four-fold increased rate of visits to the emergency department, a five-fold increased hospitalization rate, and a six-fold increased rate of nearly fatal or fatal asthma attacks [14]. As these findings show, perception represents a relevant key factor in the management of this chronic condition.

A study of Lurie [15] shows that the variables actually used by patients to determine the severity of their asthma, are very different from those that doctors employ to assess and monitor asthma severity and, in general, patients have the tendency to underestimate the level of disease severity.

The accuracy of symptom perception depends on a wide variety of cognitive and affective variables, such as emotional state, previous life experiences, attributions, contextual information, attentional and learning processes, expectation and prior asthma experiences, personality traits, and psychopathologic disturbances. All these factors can profoundly affect the perception of dyspnea and asthmatic symptoms, often independent of airflow obstruction. Many subjects with asthma learn to associate negative situations and emotional distress with difficult breathing and are thus likely to over-perceive dyspnea.

Studies in healthy volunteers and asthma patients demonstrate that subjects with high negative emotionality, tend to report more dyspnea than those with low negative emotionality. Similarly, the experimental induction of negative affective states in healthy individuals and patients in able to increase reports of respiratory sensations such as dyspnea. The feeling of unpleasantness related to dyspnea, rather than its sensory intensity, appears to be related to affective influences [16]. Individuals with asthma may under-perceive dyspnea when experiencing a positive emotional state, potentially reducing the use of prescribed medications [17].

### Alexithymia

Alexithymia, a term derived from the Greek words *alexis* (“no words”) and *thymos* (“emotion”), is a personality trait characterized. As presently defined, the alexithymia construct is composed of the following factors: (i) difficulty in identifying feelings and distinguishing between feelings and bodily sensations; (ii) difficulty in describing feelings; (iii) constricted imaginative processes; and (iv) a stimulus-bound, externally oriented cognitive style [18]. Persons with alexithymia have an impaired ability to build mental representations of their emotions, and therefore they misinterpret physical symptoms related to emotional arousal (i.e., tachycardia or tremors, dyspnea) as symptoms of somatic diseases. These subjects may increase susceptibility to disease development, therefore alexithymia is considered as a risk factor for a variety of medical conditions.

Patients with alexithymia tended to perceive and to live their disease as a cyclical disorder, not a chronic condition, and they feel high levels of distress. Furthermore, in alexithymic patients, asthma is less controlled, independent of the GINA classification of severity [6], and the lack of control leads to a reduced Health Related Quality of Life (HRQoL). Alexithymia altered the recognition of sensations and symptoms, judgments, thoughts, and emotions related to respiratory allergy. Summarizing, in asthmatic patients, the presence of alexithymia leads to report more serious negative consequences and emotions, affecting physical, psychological, and social aspects of life [19–22]. Literature concerning the links between alexithymia and asthma helps to identify some of the reasons for non-optimal disease management.

Studies focused on patients with extremely severe or nearly fatal attacks, suggest that this personality trait is associated with disease exacerbations. It has been shown that a significantly higher prevalence of alexithymia occurs among patients who have experienced a nearly fatal asthma attack (36 %), compared with patients with matched asthma severity who have not experienced a nearly fatal attack (13 %) [20] or with the general population (5–13 %). A study evaluating 270 asthmatic subjects during a severe attack showed that alexithymic patients reported significantly lower scores for nine symptom and sign categories on the Asthma Symptom Checklist scale [20]. These results show that alexithymic patients tend to underestimate both physical and emotional components of asthma exacerbations, irrespective of of the severity of the disease. A greater frequency of recurrent hospitalizations due to asthma was found in alexithymic patients (37.4 versus 28.4 % over 6 months): the difficulty in recognizing and expressing symptoms intensity and frequency could lead patients underestimate the risk of asthma exacerbation [22]. In addition, higher alexithymia scores have been found to be associated with increased report of asthma symptoms and with decreased pulmonary function, as indicated by %FEV1/FVC [23].

A recent observational study shows pulmonary function and disease control were associates to but not to anxiety and depression [24]; furthermore the level of alexithymia correlated positively with mood and anxiety.

Moreover, the presence of alexithymia has been associated with poor treatment adherence and worse HRQoL. More in detail, it was found a correlation between alexithymia and intentional non-adherence, that is the result of an active choice [25].

The prevalence of alexithymia resulted higher than in general population, indicating that it is a common feature that characterizes almost 1 patient out of 5 [26]. Despite no differences between alexithymic and non alexithymic patients were observed for what concerns GINA severity level, the level of alexithymia was associated to different disease experiences and outcomes. In alexithymic patients, asthma is less controlled, independently of GINA classification, and the lack of control leads to a poorer HRQoL.

Moreover, in asthmatic patients who develop post traumatic stress disorder (PTSD) symptoms after an asthma attack, alexithymia represents a factor that mediate the relationship between asthma severity and psychiatric co-morbidity [27].

### Coping strategies

The study of coping describe how people deal with stressful events and ongoing situations [28]. Suffering from a chronic disease such as asthma predisposes individuals to a degree of stress that requires continuous cognitive, emotional, behavioral, and social adaptation. Coping efforts may help to modify the situation, change the meaning of the experience, or mitigate the stress related to asthma management (e.g. improve asthma control, accept the disease, be optimistic about the sickness course). Active coping strategies addressed directly to the problem are considered to be adaptive, whereas passive, emotion-focused coping is related to poor adjustment to illness [28].

Studies of chronic disorders have underlined significant associations between coping styles and clinical outcomes like physical functioning, levels of disease control, morbidity, mortality and HRQoL [29]. Research suggests avoidant coping (for example, ignoring, denying, or avoiding a problem) is associated with poor HRQoL among adult asthma patients [30, 31], whereas active coping (taking an active approach to the problem, whether cognitive or behavioral) is associated with better HRQoL scores [30, 31]. Asthmatic patients with effective coping skills (characterized by maintaining a positive vision of the disease without minimizing its potential danger and by using active, cognitive strategies and flexible and diversified behaviors) will experience less psychological morbidity, a feeling of greater personal control over asthma, and better long-term management of the disease [32]. In contrast, the use of avoidance strategies is associated with a lower level of asthma control, with a worsening of clinical outcomes, and with a greater number of hospitalizations, unscheduled health care visits, and episodes of nearly fatal asthma [33, 34]. Patients with ineffective coping strategies tend to have difficult-to-treat asthma: interviews regarding behaviours to adopt during an hypothetical asthma attacks, showed that patients suffering from brittle asthma tend delayed up to 7 days before seeking medical attention [35].

Asthmatic subjects with avoidant strategy preferences are less adherent to medical therapy and prone to reliever drug abuse and to inadequate controller drug use [32]. Coping skills training and educational interventions aimed at improving coping strategies can increase a subjective sense of competence and self-efficacy in dealing with a wide range of daily demands and health issues, with benefits not only to asthma symptoms but also to psychological functioning, sense of well-being, and anxiety [36].

A large, cross-sectional survey, involving 6,474 patients and 3,089 general practitioners [29], explored coping in asthma management. Active coping strategies were described by approximately one-half of the patients, whereas the physicians reported a significantly lower frequency of effective coping strategies in their patients. A similar percentage of patients and a greater percentage of physicians reported the use of negative strategies, with “rely on fate or faith” chosen more frequently by patients than physicians. The degree of concordance between the choices of patients and general practitioners was only fair, indicating inadequate patient–physician communication.

### Depression and anxiety

Asthma has long been associated with symptoms of mood and anxiety disorders [37, 38].

Available studies show that the prevalence of anxiety and depressive disorders is more elevated among asthma patients than in general population. However, the association of mental health problems with asthma severity is controversial. Some studies have shown significantly higher level of anxiety and depression in patients with severe asthma as compared to those with milder disease, while other studies did not detect such differences [39, 40].

Clinical data has shown that the presence of psychiatric and psychological symptoms is associated with increased severity of asthma symptomatology, health service use and costs, functional impairment and poorer asthma control [41–44].

Depression would be significantly worse among severe asthmatics as compared to those with less severe asthma, and lower levels of asthma control would explain the greater degree of depressive symptoms in severe asthma. Individuals with severe asthma endorsed more symptoms of depression than those with milder asthma, with the severe asthmatics who endorsed poor asthma control being at particularly heightened risk. Psychological morbidity seems to be associated with increases in asthma severity, routine screening for depression among patients with severe asthma may result in the opportunity to tailor patient-centred clinical asthma management efforts resulting in improved disease management and control [40].

Children and adolescents with symptomatic asthma are more likely to suffer from a wide range of mental health problems, compared to healthy children. The likelihood of mental health problems appears closely associated to asthma severity. Youth with poorly controlled and/or more severe and persistent asthma may be considered a group at high vulnerability for mental health problems. Management for paediatric asthma should include screening tools and counselling interventions to detect, prevent and reduce the risk of mental health problems [40].

A study of 67 adults with severe prednisone-dependent asthma, 47 with severe non prednisone dependent and 73 patients with mild-moderate asthma [45] was aimed to investigate the prevalence of anxiety and depression symptoms. That research suggests that there are significantly more anxiety and depression symptoms in patients with severe prednisone dependent asthma compared to patients with non-steroid dependent severe asthma or mild-moderate asthma: patients with prednisone-dependent asthma were 3.5 times more likely to have clinically significant depression symptoms, and about 2.5 times more likely to have significant anxiety symptoms as compared to patients with severe or mild-moderate disease. However, the available data about the effect of steroids on mood are poor and few controlled studies have been performed to assess treatment for steroids induced psychiatric, cognitive and behavioural problems [46].

A cross-sectional study conducted at the Asthma Outpatient Clinic of the Federal University of São Paulo Hospital São Paulo [47] show how the prevalence of anxiety is higher in the patients with uncontrolled asthma than in those with controlled asthma.

In stable primary care patients, both physician and patient-reported depressive symptoms were associated to asthma severity. On the contrary, an association between depression and level of asthma control has been detected according patients’ reports, but not in those of physicians [48].

The available evidence suggests that asthma may precede and predispose to the development of anxiety and mood disorders, but also that the presence of psychological and behavioural problems may precede and predispose to asthma [49].

From a neurobiological perspective, several interesting hypotheses have been suggested with the aim to explain the association between asthma ad mood disorders: systemic inflammation seems to be a key link between these conditions and studies conducted so far suggesting a possible role of cytokines in the neurotransmission in the central nervous system (CNS) [50].

## Conclusion

The psychological characteristics of asthmatic patients and the presence of mental problems, have been shown to be linked to both asthma severity and level of disease control. Difficulties in achieving the objectives of asthma therapy suggested by current guidelines may depend also on psychological factors such as symptoms’ perception, alexithymia, coping strategies, mood disorders.

Despite numerous data support this association, a causal relationship between asthma and mental health is not clear. The hypothesis of a bidirectional influence seems to be more acceptable.

Independently of the nature of this association, when a psychological problem or difficulty is present, it interferes with an optimal disease management, especially in patients with severe asthma and poor control. At the same time, the psychological characteristics of asthmatic patients have an influence on symptoms’ recognitions, daily management and disease outcomes.

A screening of mental symptoms and psychological aspects that are known as associated to asthma, could lead to plan appropriate intervention to better control asthma and to improve the patient’s well-being.

## Abbreviations

GINA: Global initiative for asthma
ICS: Inhaled corticosteroids
LABA: Long-acting beta2-agonists
CS: Corticosteroids
ACQ: Asthma control questionnaire
ACT: Asthma control test
NAEPP: National asthma education and prevention program
ICU: Intensive care unit
FEV1: Forced expiratory volume in the first second
FVC: Forced vital capacity
HRQoL: Health related quality of life
PTSD: Post traumatic stress disorder
